# An empirical study on the reading response to picture books of children aged 5–6

**DOI:** 10.3389/fpsyg.2023.1099875

**Published:** 2023-06-13

**Authors:** Defeng Wang, Min Su, Yiyi Zheng

**Affiliations:** ^1^Weifang Engineering Vocational College, Weifang, China; ^2^Teacher Education College, Wenzhou University, Wenzhou, Zhejiang, China

**Keywords:** reading response, behavioral sequence, early reading ability, the lag sequence analysis method, picture books

## Abstract

Picture book reading has drawn a great deal of attention, while the reading response to children’s book has barely been noticed. This study therefore used the lag sequence analysis method to conduct an empirical study on the reading reaction of 60 5–6-year old children during collective picture book reading activities. Results indicated that the children had rich and diversified reading responses which mainly consisted of language description and emotional experience rather than careful observation of the picture books and in-depth understanding of the relationship between the pictures and text. In addition, the children’s oral expression and vocabulary are important predictors of differences in the reading responses of children with different reading abilities. “Image observation to personal empirical reaction” is also the key behavioral sequence that distinguishes children with different reading abilities.

## Introduction

Early reading ability, also called early literacy, refers to young children’s knowledge, skills and attitudes toward literacy that they already have before their official literacy learning begins. It also plays a significant role in young children’s language development, and even in their life-long learning (Terras et al., 2009). The ages of 3–8 is a key stage for the development of reading ability (Liu and Li, 2013). “*Kindergarten Education Guidelines (Trial)*” (Organized by the Basic Education Department of the Ministry of Education, China, 2001) clearly points out that “books, paintings and other ways should be used to arouse children’s interest in books, reading and writing, and cultivate pre-reading and writing skills.” According to a study conducted by Harvard university tracing 3–19 year-olds’ early reading abilities, early reading abilities and future study achievements are highly co-related (Snow et al., 1998). Early reading abilities can also promote children’s growth, children’s language, thinking, emotion, imagination, and so on (Zhu, 2008). Better picture-book reading ability is important to the later learning and development (e.g., Cunningham and Stanovich, 1997; Dodici et al., 2003). Furthermore, children’s reading response is a key clue for exploring the development of children’s reading abilities and its interior mechanism.

Children’s reading response is how they actively build way of expressing themselves and ways of responding to the interaction with picture books according to their previous experience. There are two main forms of response: verbal and non-verbal (Chen, 2020). The children’s reading response we focus on here actually refers to creating an environment in which diversity is respected and ideas are expressed freely. Such a response will help to promote picture-book reading activities and children’s early reading abilities with a clear direction only if importance is placed on it and it is carefully analyzed (Sipe and McGuire, 2006). Meanwhile, since each individual’s personal style, rather than simple duplication and imitation, is highly emphasized in society today, we need to respect children as independent readers, offer them opportunities to read happily, discover and investigate, support them in exploring the world poetically, and help them challenge the presupposed ideology in texts in order to cultivate their critical thinking and innovation ability (Gao and Ding, 2014).

The reader reaction theory emphasizes the centrality of the reader and how the reader itself reacts, understands and constructs its meaning with the text. Using this as a theoretical guide, our study investigated the reading response characteristics of picture books in children aged 5–6 years, and the differences in the reading response process of children with different reading comprehension abilities. Understanding these characteristics and differences can fill the gap in the existing literature, and can also provide inspiration for children’s reading education, explaining how to guide reading from children’s reading reaction, and improve children’s reading ability.

## Theoretical perspective

This study aims to explore the overall characteristics of the reading response of children aged 5–6 years, the transaction relationship between the readers and the text in the context of independent reading, and the differences in the reading response of children with different abilities, thus, just like most studies of reading responses in children (Christ et al., 2019; Chen, 2020), data collection and interpretation is guided by a theoretical perspective known as reader responses.

Reader reaction theory is the basic theory of young children’s reading reaction research Wolfgang Israel, Stanley Fish, Rosenblatt and a large number of literary theorists and critics, seriously discuss a series of issues such as readers’ reading activities, forms of text communication, and construction of personal meaning. They regard readers as active participants in the process of reading readers, believing that literature is not only an isolated text and situation written by the author, but also a dynamic interaction process between readers, text and situation. The meaning of literary works depends on the reader’s creative interpretation of the text, and the real value of the work is created by the reader. This theory is well suited for describing and explaining the process of young children’s reactions and their meaning creation in picture book reading. For example, in the reading of children’s picture books, there is not only an understanding of the basic content of picture books, but also some dramatic expressions, and even a personalized understanding combined with their own life experience, which are in line with the spiritual connotation of readers’ reaction theory. Roseblatt (1994) believes that reading is the product of the transaction of readers, texts and their literary experience, life experience, reading view and values.

In short, based on the Reader’s Response Theory, we conceptualize the response characteristics of children participating in reading. Although these children do not have the traditional literacy ability, their interaction with the text can still reflect their personalized meaning understanding and esthetic expression.

## Literature review

Early reading is the foundation of lifelong learning. In recent years, more and more attention has been paid to early reading in the field of Chinese children’s education, and many supporting policies have been released. The Outline for The Development of Chinese Children (2021–2030) points out that the review of children’s publications should be strengthened, and excellent children’s books should be recommended by age. There are also many early reading practices. For example, more and more kindergartens begin to be equipped with reading function rooms, children’s picture books are becoming more and more common, and more and more people are participating in reading promotion activities. However, reading response-related studies are still relatively limited. Based on reader response theory, we reviewed research on young children’s reading response and the relationship between reading response and reading ability.

### Children’s reading reaction

For the definition of reading reaction, most scholars (Jalongo, 2004; Chen, 2020; Su, 2021) believe that reading reaction is the reaction to the whole reading process. For example, Chen (2020) follows the classification of children’s reading reaction in Jalongo, defining the reading reaction as the five categories of action, micro-expression, esthetic expression, language and emotional reaction shown in the picture book reading of children with intellectual disabilities. However, some scholars believe that children’s reading reaction is mainly oral reaction. For example, Zhang (1993) pointed out that readers’ reaction to literary works includes four categories: integration, description, interpretation and evaluation. In addition, some researchers have also tried to explore the internal influencing factors of children’s reading reactions to reading picture books. Internally, including children’s age, gender (Stone, 1984), children’s reading ability, literacy ability, cognitive ability (Xie, 2007), stereotypes, temperament and personality (Li, 2015), etc. From the external level, including teachers’ professional quality and support (He, 2003), the characteristics of the picture book itself, life experience, social background, and the influence of researchers (McClung, 2017), etc.

However, the number and distribution of studies describing the overall characteristics of young children’s reading reactions are limited. We found that (Sipe and McGuire, 2006) after observing children’s collective reading, group reading, and one-to-one reading for 7 months, the five manifestations of children’s reading response are analytical, intertextuality, individual, transparency and expression. Among them, the analysis accounted for the largest proportion (73%), while the related life experience accounted for the lowest proportion (2%). Meanwhile, he also proposed that children’s reading response is personalized. Braid and Finch (2015) developed the model system of Sip and proposed the thinking type of literary reaction from simple to complex, but without further describing the overall characteristics of children’s reading reaction. However, Chinese scholars’ research on reading response characteristics focuses more on oral response (Lin and Zhou, 2012; Li, 2015), while the research on verbal response is still insufficient, We have found only two articles: Lin (2002) made a comparative study on the reading reaction of senior class children when reading e-books and paper picture books. The study found that the oral reaction of children watching electronic children’s books appeared: expressing their preferences, questions about the story, questions about the form of electronic media expressing them, predicting the plot, retelling the sentences in the film, describing the pictures in the film, and connecting with their own experience. Body reaction: finger screen, smile, open the mouth, imitate the action of the character in the film, hold the body or hand to the CD, and express their opinions with the action. Picture reaction: presents the characters, scenes and children’s life experience in electronic children’s books. Chunmei (2008) on the reading reaction of small class children found that 3-year-old children showed rich reader reactions, pointing to the illustrations and telling the illustration as the main clue. In addition, reaction behaviors to picture books are intertwined with the game. The above two studies described the categories of reading reaction characteristics, but did not further analyze the different response categories, especially the proportion, distribution and the relationship between the various response categories. Extending the correlation study, we explored the overall distribution of reading responses in young children based on behavioral sequence analysis, and successively analyzed the correlation between various reading response categories.

### The relationship between children’s reading reaction and reading ability

According to previous studies on the influencing factors of reading reaction, there is a positive correlation between children’s reading reaction status and reading comprehension results (Christ et al., 2019). At the same time, it is worth noting that some studies show that teachers’ support for reading reaction will also affect and promote the improvement of reading ability (Boyd and Maloof, 2000; Kim, 2004; Amos, 2008). Su (2021) pointed out that reading reaction is an important clue to explore the process of children’s reading comprehension. There is a close connection between children’s reading reaction and its reading content. Children’s language and behavior ability participate in the process of meaning construction, and affect children’s feelings, understanding and expression in the reading process. Aihua (2014) also explores the reading reaction of children’s picture books from the reader reaction theory, and advocates improving the reading ability of children’s picture books by exploring children’s reading reaction. Therefore, understanding and analyzing children’s reading reaction and grasping the overall state of children’s reading are the necessary prerequisite for analyzing and exploring children’s reading ability. To sum up, the relationship between reading response and reading ability is to influence and promote each other. Therefore, in this study, the researchers measured the differences in children’s reading responses at different reading ability levels to provide reference for preschool teachers to support activities for children’s reading responses.

The above related studies have given us a gradually clear understanding of children’s reading response and its correlation with reading ability, but it is not difficult to find that the current relevant research is still relatively limited. First, the number and distribution of the reader’s response studies in children’s picture books are relatively limited. Although researchers have conducted a series of studies on the concept, characteristics and influencing factors of reading response, the overall characteristics of reading response and the correlation between subcategories still need to be explored. Secondly, the design and measurement are still simple, lacking a unified measurement standard. Although some studies used eye movement experiments and questionnaires to assist qualitative research (Zheng, 2011; Chen, 2012), however, most of them are still qualitative studies, and only one relevant quantitative study is found (Christ et al., 2019). Furthermore, the current research mainly consists of qualitative studies. Third, the study content is still too horizontal since it places more emphasis on verbal responses, while paying much less attention to other non-verbal responses. Therefore, this study aimed to investigate 5–6 year-old children’s reading responses during collective reading, and to compare different reading responses of children with different reading abilities. Furthermore, based on the study findings, educational suggestions will be proposed to draw more attention to children’s reading responses in the academic field and finally to provide a reference and inspiration for related studies and teaching in the future.

Therefore, this study examined the reading response of young children aged 5–6 years in picture book reading. It is guided by two research questions.

RQ1. In picture book reading, what is the distribution of reading reactions of the sampled children and what are the overall characteristics?

RQ2. In picture book reading, what are the differences in the reading responses of children with different levels of reading ability?

## Research methods

### Research participants

Five to 6-year-old children, in the key stage of moving from kindergarten to primary school, have increasingly mature cognizing abilities, thinking patterns and strong desires to read. Therefore, this study adopted random sampling to select 60 5–6-year-old children from C kindergarten, in Wenzhou, Zhejiang as research participants. The kindergarten is public-owned and attached to a university. After obtaining the consent of the kindergarten principal and teachers, the researchers explained the purpose of the study to the children and the parents of the kindergarten. We sent the parental consent form to the parents. Finally, a total of 60 children agreed to participate in the study. The mean month age of the participants was 67.42 (SD = 3.581), including 31 boys (mean month age 67.58, SD = 3.745) and 29 girls (mean month age 67.32, SD = 3.581). According to the self-reports of young children and their parents, as well as the confirmation of teachers, the subjects had similar reading education experiences and experiences.

### Research tools

#### Assessment of early reading abilities

This research conducted a reading ability test, referring to the study by Christ et al. (2019). First, we adopted the CPA test proposed by Clay (2002); this test assesses how children sense books, including the books’ covers, back covers, title pages, how spoken language and movements correspond to the books’ content, reading direction, key details, picture and words. The children get one score when they mention one element. Second, the test also assesses children’s reading comprehension via retelling, content comprehension and creativity. The retelling part here is assessed according to four elements: characters, problems, issues and solution; the content comprehension is conducted from six dimensions: the characters’ status, the story’s details, problem-solution, emotions and feelings, and the story’s themes. Third, post-reading expression was assessed by inferential and critical problems. Each element is scored from 0 to 2, with 2 for 100% right, 1 for partly right, and 0 for wrong or no answer. The reading material used in this research was the Kate Greenaway Medal-winning *Mr. Gumpy’s Outing*, which is suitable for children at this age. The test discussed here has been reviewed by experts and teachers in the pre-school language field and has been pre-tested and modified many times. Its internal consistence is 0.796. Children’s reading ability test is mainly in leisure time and corner activity time, while children participate in one-on-one tests in the reading function room. The specific test procedure will be introduced in the “Research Procedure.”

#### Reading-response coding scheme

In order to investigate and analyze the current situation of children’s collective reading reaction, we compiled Reading-response coding Scheme and conducted related sequence analysis. To guarantee the reliability of the behavior analysis，two researchers performed the coding in two stages. In the first stage, referring to Kiefer (1995), the children’s reading responses were divided into five categories and 13 codes (see Table 1). Then, to make the coding scheme sufficiently valid, three experts on picture-book reading were invited to ensure that the codes could be adapted to the corresponding elements. In the second stage, the researchers coded the reading responses recorded in videos based on the coding scheme, with 828 reading responses coded in total. As a result, this test proved to be highly reliable with its 0.898 double-coding internal consistency.

**Table 1:** Codes for children’s reading response.

Categories	Code	Element	Description
Visual sensation (S)	SW	Watching words	Pay attention to words and watch them for a while
	ST	Watching pictures	Pay attention to pictures and watch them for a while
	SS	Watching peritext	Paying attention to and watch peritexts for a while
Interaction (X)	XZ	Identifying and cognizing	Identifying pictures and words, finding the corresponding ones
	XX	Role playing	Making actions and role playing according to the book’s content
Language Description (Y)	YW	Word description	Describing the shape and meaning of words
	YT	Picture description	Describing the scenes, characters, actions and plots in the pictures
	YY	Relevance description	Describing how the meaning of the pictures and words relate to each other
Emotion expression (Q)	QQ	Emotion expression	Expressing how they experience the characters and plots and whether they feel happy or sad, etc.
	QG	Personal-experience response	Children will intentionally relate their life and text-reading experience
	QF	Analysis and evaluation	Making analysis, evaluation, moral judgment, and so on
Esthetic performance (M)	MC	Creative expression	Esthetic experience and boundless imagination such as imitation, or even creating things
	MY	Artistic response	Children giving artistic responses relying on pictures, music, handcraft, construction, and so on.

### Research procedure

The first step was assessing the children’s reading ability. With permission from the head teacher and teachers in the kindergarten, the children were tested by trained postgraduates majoring in pre-school education. Before the test, the leading testers familiarized themselves with the children in their classes. According to the existing learning plan and children’s interests, the book Mr. Gumpy’s Outing was provided to children, and to ensure that every child had never read the book. The test, used the children’s corner activity time and leisure time，conducted in the kindergarten’s reading room, lasted for 2 weeks and started with, “Today, the teachers bring all of you a really interesting picture book. Could read this book and tell me the story in it?.” After that, the leading tester asked the children some questions according to the pre-designed question outline.

The second step was observing and recording the collective reading. To fully capture the reading response of young children and facilitate later sequence analysis of videos, the 60 children were divided into four groups and participated in the same reading activities instructed by the same teacher. The camera was used to video the entire course of the reading activity.

### Statistics and analysis

For the reading ability test, the leading testers completed a training session and a trial test before the formal test. On the day of the formal test, the leading testers conducted the test according to the test scheme. After the test, the testers watched and then scored the videos based on related standards, and used SPSS25 (a statistic software package) to perform the statistical analysis. In the reading-response coding part, two trained postgraduates in pre-school education major used ELAN (a video analyzing software package), used the back-to-back method, based on the reading reaction coding scheme (see Table 1), to code the reading sharing activity videos collected in the early stage, so as to determine the type of ongoing reading reactions of children. An expert was invited to supervise and check the whole process. Then, any disagreements between testers and experts were discussed until consensus was reached. Sampling the videos’ code is as precise as 0.01 s, and then the sequence analysis was generated by GSEQ5.1 (a sequence analysis software package) to explore the correlation between various reading reactions.

## Results and analysis

### Analysis of 5–6-year-old children’s reading abilities

According to the total score of the 60 5–6-year-old children’s reading abilities, the test participants’ scores ranged from 6 to 25. The reading abilities of most children were at the intermediate level since most of the children scored 15–16, while only a few had low scores and one scored full points. That is to say, their reading abilities still have large room to grow. Besides, the approximately normal distribution with 0.309 skewness and 0.194 kurtosis shows that the tools used here to test the children’s early reading abilities are reasonable (Table 2).

**Table 2:** Frequencies of codes for the children’s picture-book reading responses.

	Reading response													
Categories	Visual sensation (S)			Interaction (X)		Language description (Y)			Emotion expression (Q)			Esthetical performance (M)		
Codes	SW	ST	SS	XZ	XX	YW	YT	YY	QQ	QG	QF	MC	MY	Total
Collective reading	1	1	0	2	158	20	232	0	61	64	117	81	91	828
	0.1%	0.1%	-	0.2%	19%	2.4%	28%	-	7%	8%	14%	10%	11%	

### Analysis of 5–6 children’s reading response features

#### Frequency analysis

The frequency analysis of the 828 codes of reading responses from the 60 children in the collective reading activities is presented in Table 3.

**Table 3:** Code frequencies of the high reading ability level students.

	Reading response													
Categories	Visual Sensation (S)			Interaction (X)		Language description (Y)			Emotion expression (Q)			Esthetical performance (M)		
Codes	SW	ST	SS	XZ	XX	YW	YT	YY	QQ	QG	QF	MC	MY	Total
Collective reading	0	1	0	1	87	20	140	0	26	46	86	56	42	505
	-	0.2%	-	0.2%	17%	4%	28%	-	5%	9%	17%	11%	8%	
	0.20%			17.20%		32%			31%			19%		

**Figure 1:**
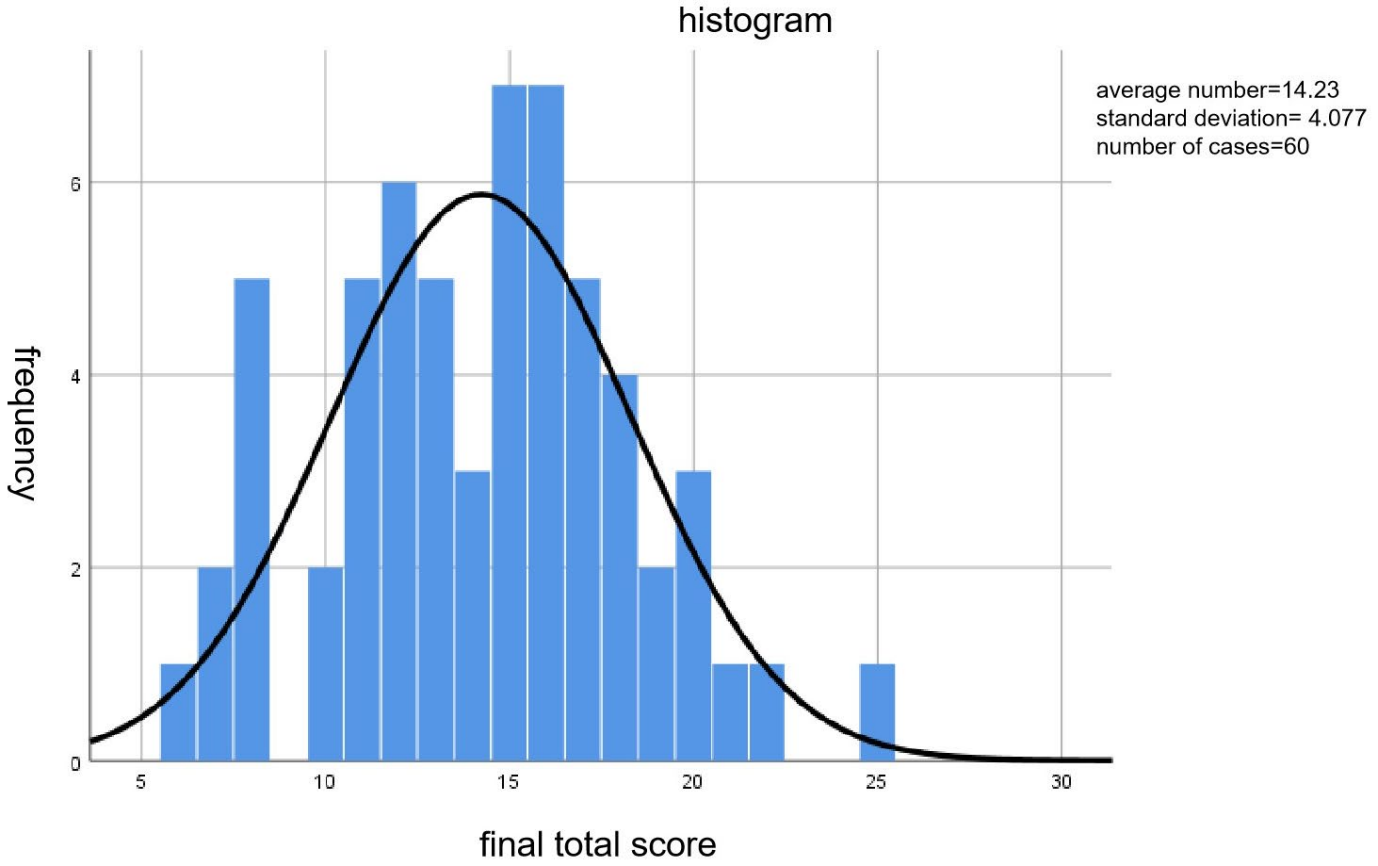
Distribution of reading-ability scores.

In general, the frequencies of the five reading responses are not distributed evenly: language description (Y, 30.4%), emotion expression (Q, 29%) account for more, next are esthetical performance (M, 21%) and interaction (X, 19.2%), while visual sensation (S, 0.2%)is the least. Specifically, picture description (YE) is the most, followed by role play (XX), analysis and evaluation (QF), artistic response (MY), creative expression (MC), emotion expression (QQ), personal-experience response (QG) and word description (YW), while Identifying and cognizing (XZ), watching peritext (SS), relevance description (YY), watching words (SW) and watching pictures (ST) all counted for less, or even did not appear at all. This result indicates that in collective reading, 5–6-year-old children’s reading response mainly focused on the language response and emotional experience of pictures, and they can interact with books verbally, and with body language, artistic performance, and so on, but they lack detailed observation and deep understanding of picture books (Figure 1).

#### Sequence analysis

**Figure 2:**
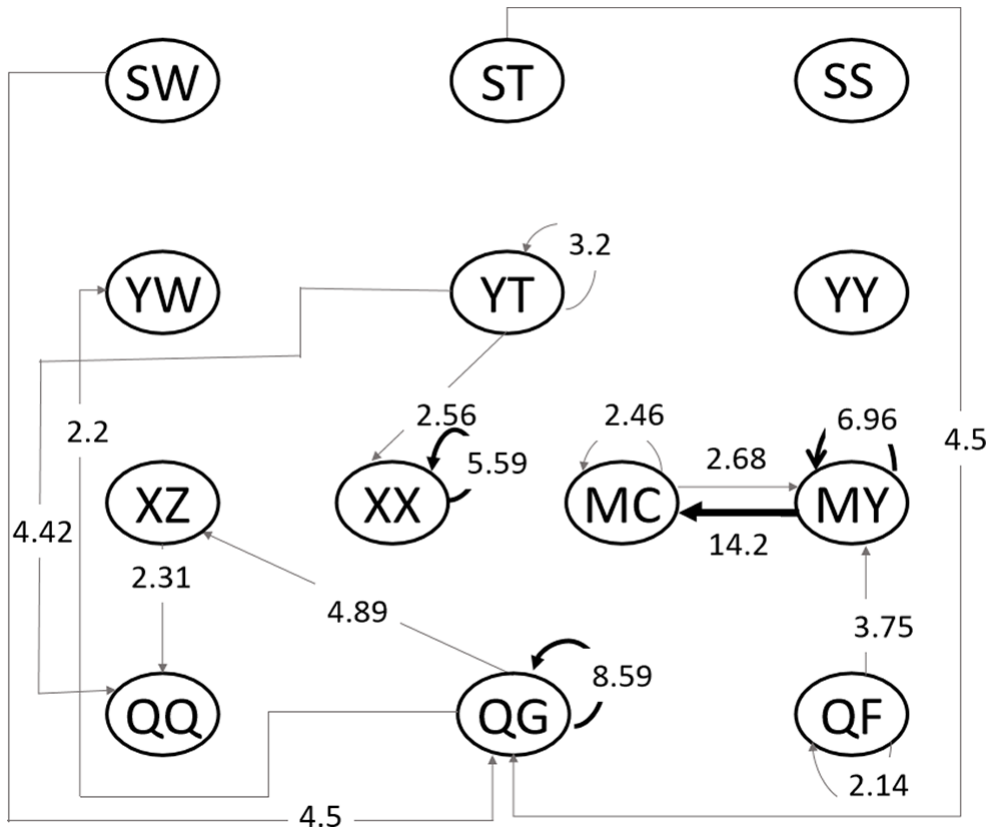
Behavioral sequence diagram. SW, watching words; ST, watching pictures; SS, watching peritext; XZ, identifying and cognizing; XX, role playing; YW, word description; YT, picture description; YY, relevance description; QQ, emotion expression; QG, personal-experience response; QF, analysis and evaluation; MC, creative expression; and MY, artistic response.

For further analysis of participants’ reading-response modes, all reading-response codes were input into GESQ5.1 to generate a residual plot. If Z > 1.96, this behavioral sequence is regarded as reaching the significant level. Then we transfer the residual plot into a behavioral sequence diagram, as shown in Figure 2. The arrow shows the direction of the significant sequence, the number represents the value of Z, and width of the lines shows the level of action-sequence significance.

From Figure 2, it can be seen that there are 16 significant reading-response sequences in collective reading and numerous links among the various codes. In general, the children’s reading response shows three independent modes: (1) children prefer to connect what they read with their personal experiences (QG) after watching pictures (ST) and watching words (SW), while children with literacy experience may describe words (YW), otherwise they will do cognizing (XZ) and emotion expression (QQ); (2) children love continuously making analysis and evaluation (QF) in order to generate creative expression (MC); (3) children will also do picture description (YT) in order to role play (XX) or for emotion expression (QQ). From the above-mentioned, the children’s reading responses were diversified and they will express or even create things with their own experiences based on how they sense and understand.

### Reading-response comparison of high- and low-reading ability level children

#### Frequency analysis

In order to compare the reading responses of different participants, the 60 participants were divided into high and low reading ability levels. The top 50% were categorized as the high reading-ability level while the remainder were in the low level. The code frequencies of the two groups are shown in Tables 3, 4.

**Table 4:** Code frequencies of the low reading ability level students.

	Reading response													
Categories	Visual sensation (S)			Interaction (X)		Language description (Y)			Emotion expression (Q)			Esthetical performance (M)		
Codes	SW	ST	SS	XZ	XX	YW	YT	YY	QQ	QG	QF	MC	MY	Total
Collective reading	1	0	0	1	71	0	92	0	35	18	31	25	49	323
	0.3%	-	-	0.3%	22%	-	28%	-	11%	6%	10%	8%	15%	
	0.30%			22.30%		28%			27%			23%		

As shown in the tables, the total number of reading responses from the high reading ability level students is 1.56 times that of the low level students. This indicates that the higher level group had a greater variety of reading responses. Furthermore, among the five reading responses, only the visual sensation frequency of the two groups was the same, with both groups equal to 1. For the other four responses, the number of the higher level students is greater than that of the lower level students. For example, the frequency of interaction (X), language description (Y), emotion expression (Q) and esthetic performance (M) of the higher level group are, respectively, 1.22, 1.74, 1.88, and 1.32 times that of the low level group. Specifically, the high reading ability group tended to engage in more analysis and evaluation (QF, 17%) and creative expression (MY, 15%). To sum up, the reading responses of the high reading ability group are more varied, while their visual sensation is normal. At the same time, the lower level students showed more diversified creative expressions and emotion experiences even though they were weaker in content comprehension, expression and critical thinking.

#### Sequence analysis

**Figure 3:**
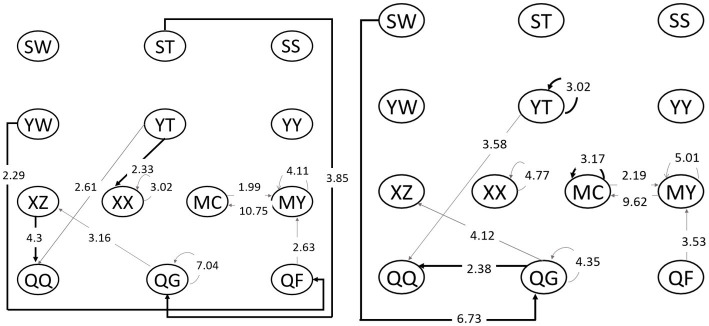
Behavioral sequence diagram. High reading-ability group (left); Low reading-ability group (right). SW, watching words; ST, watching pictures; SS, watching peritext; XA, identifying and cognizing; XX, role playing; YW, word description; YT, picture description; YY, relevance description; QQ, emotion expression; QG, personal-experience response; QF, analysis and evaluation; MC, creative expression; and MY, artistic response.

Figure 3 shows the behavioral sequence diagram of the high and low reading ability groups. The values marked on each line represent the Z value of the corresponding sequence and the line’s direction shows which direction such action transfers to. The gray line means the reading responses behavioral sequence of two groups in collective reading, while the dark lines shows the sequences only presented in one group.

In collective reading, differences in the reading response of the two groups mainly focus on: (1) for different word-cognizing ability, the lower group has emotion expression (QQ) connected with their personal-experience response to deliver word description when watching words (SW) was difficult for them, while the higher group could generate word description even when making analysis and evaluation of the words they read. (2) When role playing, the higher level students could role play (XX) based on the picture description (YT) while the lower group could not connect with other responses. (3) The higher group watched pictures (ST) carefully and made expressions based on their personal experiences (QG), which could not be found in the lower level group. That is to say, the children’s reading responses from high reading ability works more fluently, and they could analyze, judge and express their emotions with their personal experiences and the book content.

## Discussion

Our study extends the previous work in several ways. First, we expanded the limited study of the overall characteristics of children’s reading responses (Styles and Arizpe, 2001; Su, 2021) to explore the multi-dimensional characteristics of reader reading responses including oral and non-spoken responses through frequency and sequence analysis. Second, compared to previous studies, we explored new variables, exploring differences in balance reflection among children with different reading abilities in the analysis. Finally, and most importantly, we pay attention to the reaction process in children’s picture book reading, which expands the research on early children’s reading (Sofologi et al., 2022; Sucena et al., 2023) and brings new inspiration to the improvement of children’s reading ability and reading teaching. In the following sections, we will discuss the overall characteristics of young children’s reading reactions, the differences in children with different reading abilities, and discuss the implications.

### The overall features of the 5–6-year-old children’s reading responses

According to this research, the 5–6-year-old children had various reading responses. They usually described pictures and responded with word description and emotional expression in various ways. They also constructed responses with artistic performance and role playing on the basis of their own experiences. Children’s cognition mainly consists of concrete thinking, which is concrete, multi-dimensional and dynamic, and needs to be completed by their sensorimotor ability (Eisenke and Keane, 2002). For example, when reading the phrase “invite chicken to join his boat ride” from *Mr. Gumpy’s Outing*, the teachers asked, “What is it?,” and C answered “a chicken” and imitated a cock crowing at the same time. He also said, “The bigger one has a rainbow on it while the little one is the baby!” with his arms open, imitating chick’s movements and telling the teacher that this is how chicks act. Another example, M happily pointed to Mr. Gumpy in the reading and said, “He is wearing a straw hat, this must be farmer, I have seen a farmer is also wearing a straw hat before.” Roseblatt (1994) also stated that readers may analyze text esthetically, which is shown by the emotional connection in the interaction between the reader and the story. According to Jauss (1982), the key concept of accepting esthetics is “horizon of expectations,” the so-called horizon expectations is the thinking orientation or first structure of readers’ literary experience, life experience, reading view and values.

Therefore, C said that the chicken is the child of the big chicken, and M said that Mr. Gumpy was a farmer, all based on their life experience.

However, it was found that in collective reading, children fail to have enough detailed observation of illustrations, words, peritexts and deep understanding of how pictures and words connect with each other. There are a number of factors that may contribute to this. First, teachers themselves do not have enough detailed observations of children’s picture books nor carefully designed guidance. Based on interviews after the study, some teachers think that they find it difficult to deeply understand picture books, which has also been found in previous studies. According to Sipe and McGuire (2006), teachers generally fail to pay enough attention to the front and back covers，endpapers and dedication page, even when they notice these parts. Secondly, the traditional reading-teaching mode is too rigid to effectively instruct children. It has already been found that the traditional teaching mode is better for making texts’ gist and content understood within a short period of time for big classes with many children, but that the children hardly have the chance to discuss and share with each other since they are not given enough time (Vezzani, 2019). Mercer and Dawes (2008) also pointed out that longer waiting time can give students more space to think, and then gain greater learning benefits. Thirdly, since view on children is the foundation of the concept of education, it is difficult for teachers to change their role from being the sole authority in reading classes, so children will be marginalized in the class. Picture books include complicated relations between pictures and books. Meanwhile, other peritexts such as the front and back covers and title pages are all key to the scene setting, and provide clues and hints about the story. According to Doonan (1993), children need more time to have “close observation” of picture books in order to find authors’ clues and hints to fill in the blanks proposed by Wolfgang (1991) and to create extra meaning.

In conclusion, the general features of the reading response of 5–6-year-old children’s collective reading are as follows: for what to read, children will mainly look at the pictures, while reading the words is just a supplement; as for how to read, children will mainly rely on oral language and emotional experiences with interaction and esthetic performance as a supplement. However, they fail to observe picture books carefully.

### Differences between reading responses from 5 to 6-year-old children’s high and low reading-ability

By comparing reading-response frequencies of high and low reading ability children, it was found that in all categories, except for visual sensation, for which both groups showed less frequency, the higher level group has more various frequencies, which indicates that reading abilities will influence reading responses to some extent. Among the previous studies such as the investigative report on the reading response of a child called “Hull,” Hunt (2005) proposed that the more mature the reading is, the more reading modes children will have. Monson and Howe (1991) also connected the taxonomy of reading comprehension with all kinds of reading responses in order to prove that the parallel levels between levels of comprehension and reading response are the same as each other. It is also worth noting that the results of visual sensation are different from those of previous studies, since children from the high reading ability group did not show any obvious advantage over the lower level group. This finding is consistent with the above-mentioned research result that children’s reading response lacks detailed observation of pictures and words. This also shows that it is really necessary for teachers to better instruct children to observe picture books.

In the behavioral sequence comparison, there were also some differences in the reading responses of the two groups:

First, the high reading ability group made analysis and evaluation based on the text itself after their word description much more easily, while the lower level group showed their various understandings and personal expressions such as emotion expression and identifying and cognizing connected with their personal experience when they have difficulties in describing. Previous studies have shown that the more readers integrate into reading, the more they tend to make literary judgments (Hunt, 2005). In addition, the results of this study also emphasize that literacy is an important factor in distinguishing the reading response patterns of children in the high and low reading ability groups. Biemiller (1999) has pointed out that recognizing words and understanding text are the key to successful reading. Children in the low level reading ability group often expressed something contrary to the original meaning since they had problems identifying the written words. For example, D from the lower level group pointed at the sheep in the book and said, “This is Pleasant Sheep (a character from a Chinese cartoon)” but she was immediately corrected by her teacher. There is no doubt that the teacher’s reaction was reasonable to some extent, but she also suppressed D’s desire to express herself. According to Harold Bloom, such misreading is a part of readers’ creative strategies and is a unique way for children to acquire meanings instead of misunderstanding content (Bloom, 1980). Even though D’s interpretation was not what the author wanted to express, D still achieved logical self-consistency and meaning creation based on his own literal experiences.

Second, the role playing of the low level reading ability group formed its own independent closed loop, while that of the higher level group connected closely with the picture descriptions. That is to say, the verbal description of the pictures is also a key factor distinguishing the reading response of the higher level group from that of the lower level group. Meanwhile, some tracing studies also show that children who have high verbal-expression ability in their kindergarten stage will easily experience achievements in reading learning after entering elementary school (Catts et al., 2006). The reasons why oral language and words can be factors distinguishing the high reading-ability group from the lower level group are as follows: cognizing, understanding and expressing the pictures and words in picture books is the first step of learning to read, and is also the accepting step. If children face some obstacles in this step, it will be difficult for them to sense, understand and express. Therefore, the ability to code pictures and words in picture books can measure children’s early reading ability. According to *Pathways to reading: the role of oral language in the transition to reading,* children’s words accumulation in oral language can help them connect phonetics and semantics in order to promote reading ability (Belsky et al., 2005).

Third, children from the high ability group carefully observed pictures and made expressions based on their personal experiences, which was a less common behavioral sequence in the lower group. This indicates that this is the key sequence distinguishing the two groups. Rosenblatt (2009) proposed a concept called active readers who will draw on a reservoir of their past experience to interpret stories from books when they read and discuss with others. At the same time, reading responses also include the readers’ emotional connection with books during interaction between these two. Children from the high reading ability group have these features of such active readers.

In conclusion, the main differences between the 5–6-year-old children’s high and low reading-ability groups are as follows. First, the children’s reading responses were relevant to their reading abilities to some extent, while the higher group did not show any advantages in the visual sensation dimension. Second, oral language and words are key factors distinguishing the reading responses of the high and low reading ability groups. The higher group tended to make analysis and judgment, while the lower group prefer diversified ways of expressing themselves. Besides, “observation and personal empirical reaction” are also the key sequence to distinguish the two groups.

## Limitations and future research directions

The study object and study design may limit the study in three aspects: (1) The study object selected by this study is C kindergarten of public kindergarten, Wenzhou city, and more other types of kindergartens were not studied. Follow-up studies can expand the scope in breadth, and further try to choose more types of kindergartens or other age classes for children’s reading response. (2) This study adopted the behavioral sequence analysis method. The coding in the analysis limited the number of participants we can include in the data. Although the behavior and coding volume were large, the small number of participants limited the universality of the research results. Future studies may expand this work. (3) Based on the perspective of reader response theory, this study is committed to exploring children’s reading response and the differences in children’s reading response at different reading ability levels. The improvement of children’s reading ability is a long process, so the research on children’s reading reaction and support is inevitably a long-term action. In the future, we can further discuss how to support the reading reaction based on children’s different reading ability.

## Conclusion

In conclusion, this study highlights the need to respect children’s reader status, observe, understand, and support children’s diverse reading responses. This study found that young children have rich reading reactions, mostly with language description and emotional experience, and their deep understanding of picture books needs to be strengthened.” Image observation-personal empirical response” is the key behavioral sequence of reading responses that distinguishes the level of reading ability. Word and oral expression are one of the important factors affecting the difference in reading response patterns of children with different reading abilities.

From a practical point of view, this inspires us that mature reading expression is an interactive process based on sufficient image observation and integrating observation with personal knowledge experience and life experience to establish personalized graphical representation of knowledge experience. So reading teaching should be given enough “observation time” on the basis of, pay attention to observation, analysis and support children’s reading reaction, fully mobilize children’s own social and cultural experience to stimulate their interest in reading, at the same time cannot ignore other types of reading description, pay attention to multimodal expression, gradually realize the purpose of depth reading.

## Data availability statement

The original contributions presented in the study are included in the article/supplementary material, further inquiries can be directed to the corresponding author.

## Ethics statement

The studies involving human participants were reviewed and approved by the Life Sciences and Research Ethics and Safety Committee of the University of WenZhou. Written informed consent to participate in this study was provided by the participants’ legal guardian/next of kin.

## Author contributions

DW: research design, data collection, data interpretation, and writing the main manuscript text. MS and YZ: data collection and data interpretation. All authors contributed to the article and approved the submitted version.

## Funding

This work was supported by the Project of the 13th Five Year Plan of Educational Science in China (EHA190497).

## Conflict of interest

The authors declare that the research was conducted in the absence of any commercial or financial relationships that could be construed as a potential conflict of interest.

## Publisher’s note

All claims expressed in this article are solely those of the authors and do not necessarily represent those of their affiliated organizations, or those of the publisher, the editors and the reviewers. Any product that may be evaluated in this article, or claim that may be made by its manufacturer, is not guaranteed or endorsed by the publisher.

## References

[ref1] Aihua G. (2014). Rereaction of primary school students reading picture books (Master's dissertation). Shanghai: Shanghai Normal University.

[ref2] Amos P. (2008). The role of literature in instructed foreign language learning and teaching: an evidence-based survey. Lang. Teach. 41, 465–496. doi: 10.1017/S026144480800520X

[ref3] Belsky J. Booth-LaForce C. L. Bradley R. Brownell C. Burchinal M. Campbell S. B.. (2005). Pathways to Reading: the role of Oral language in the transition to Reading. Dev. Psychol. 41, 428–442. doi: 10.1037/0012-1649.41.2.428, PMID: 15769197

[ref4] Biemiller A. (1999). Language and reading success, vol. 5. Cambridge, Mass: Brookline Books.

[ref5] Bloom H. (1980). A map of misreading. Oxford City, England: Oxford University Press.

[ref6] Boyd M. Maloof V. M. (2000). “How teachers can build on student-proposed intertextual links to facilitate student talk in the ESL classroom” in Second and foreign language learning through classroom interaction. eds. Hall J. K. Verplaetse L. S. (Mahwah, NJ: Erlbaum), 163–182.

[ref7] Braid C. Finch B. (2015). "ah, i know why … ": children developing understandings through engaging with a picture book. Literacy 49, 115–122. doi: 10.1111/lit.12057

[ref8] Catts H. W. Adlof S. M. Weismer S. E. (2006). Language deficits in poor Comprehenders: a case for the simple view of Reading. J. Speech Lang. Hear. Res. 49, 278–293. doi: 10.1044/1092-4388(2006/023), PMID: 16671844

[ref9] Chen Y. Z. (2012). Study on the children's reaction to reading Leo oni's allegorical picture books. (Master's Dissertation). Chiayi: Chiayi University.

[ref10] Chen X. C. (2020). Field study of the Reading reaction of picture books in children with intellectual disabilities (Ph.D. Dissertation.). Huaibei: Huaibei Normal University.

[ref11] Christ T. Wang X. C. Chiu M. M. Cho H. J. E. (2019). Kindergartener’s meaning making with multimodal app books: the relations amongst reader characteristics, app book characteristics, and comprehension outcomes. Early Child Res. Q. 47, 357–372. doi: 10.1016/j.ecresq.2019.01.003

[ref12] Chunmei L. (2008). Reading is a process of social interaction: take the reaction of small class children to picture books for example. Min. J. 34, 281–296. doi: 10.29688/mhj.200802.0018

[ref13] Clay M. M. (2002). Observational survey (2nd. Edn.). Portsmouth, NH: Heinemann.

[ref14] Cunningham A. E. Stanovich K. E. (1997). Early reading acquisition and its relation to reading experience and ability 10 years later. Dev. Psychol. 33, 934–945. doi: 10.1037/0012-1649.33.6.934, PMID: 9383616

[ref15] Dodici B. J. Draper D. C. Peterson C. A. (2003). Early parent—child interactions and early literacy development. Top. Early Child. Spec. Educ. 23, 124–136. doi: 10.1177/02711214030230030301

[ref16] Doonan J. (1993). Looking at pictures in picture books. Urumqi, China: Xinjiang Youth Press.

[ref17] Eisenke A. Keane S. (2002). Cognitive psychology. Shanghai, China: East China Normal University Press.

[ref18] Gao J. P. Ding G. G. (2014). From the study of literary and artistic psychology to the theory of the reader's reaction (in Chinese). Anhui, China: Anhui Literature and Art Press.

[ref19] He Y. J. (2003) Study on the meaning construction of children's reading of picture books (Ph.D. Dissertation.).Chiayi: Chiayi University.

[ref20] Hunt P. (2005). Understanding children's literature: Key essays from the international companion encyclopedia of children's literature. London, UK: Routledge.

[ref21] Jalongo M. (2004). Young children and picture books. 2nd Edn. New York, USA: National Association for the Education of Young Children, 179.

[ref22] Jauss H. R. (1982). Aesthetic experience and literary hermeneutics. Nisu State Twin City, USA: University of Minnesota Press.

[ref23] Kiefer B. (1995). The potential of picture books from visual literacy to aesthetic understanding. Englewood Cliffs 6, 40–43.

[ref24] Kim M. (2004). Literature discussions in adult L2 learning. Lang. Educ. 18, 145–166. doi: 10.1080/09500780408666872

[ref25] Li P. X. (2015). The study of oral reaction and discussion interaction between children reading picture books. Educ Acad J 7, 113–140.

[ref26] Lin W. L. (2002). Investigation and response of picture books and electronic children's books in Taipei city children (Ph.D. Dissertation.). Taipei: Taiwan Normal University.

[ref27] Lin H. J. Zhou W. X. (2012). “How to read it without words?”: children read the image language in a picture book without words. Contempor Educ Res Instit Invest 9, 1–37. doi: 10.6151/CERQ.2012.2003.01

[ref28] Liu B. G. Li L. H. (2013). Early reading concept and picture book reading teaching. Res Preschool Educ 7, 55–60.

[ref29] Mercer N. Dawes L. (2008) Exploring talk in school: Inspired by the work of Douglas Barnes. London: Sage, 55.

[ref30] Monson D. L. Howe K. (1991). Trade books and the social studies curriculum. Publ. Res. Q. 7, 37–46. doi: 10.1007/BF02678159

[ref31] McClung N. A. (2017). Learning to queer text: epiphanies from a family critical literacy practice. Read. Teach. 71, 401–409.

[ref32] Organized by the Basic Education Department of the Ministry of Education, China (2001). Kindergarten Education Guidance Outline (trial) Interpretation. Nanjing, China: Jiangsu Phoenix Educational Press.

[ref33] Roseblatt L. M. (1994). The reader, the text, the poem: The transactional theory of the literary work. Illinois, United States: Southern Illinois University Press, 45.

[ref34] Rosenblatt L. M. (2009). The literary transaction: evocation and response. Theory Pract. 21, 268–277. doi: 10.1080/00405848209543018

[ref35] Sipe L. R. McGuire C. E. (2006). Picturebook endpapers: resources for literary and aesthetic interpretation. Child. Lit. Educ. 37, 291–304. doi: 10.1007/s10583-006-9007-3

[ref36] Snow C. E. Burns M. S. Griffin P. (1998). Preventing reading difficulties in young children. Washington, DC: National Academy Press.

[ref37] Sofologi M. Papatzikis E. Kougioumtzis G. Kosmidou E. Klitsioti A. Droutme A.. (2022). Effectiveness of musical training on Reading comprehension in elementary school children. Is There an Associative Cognitive Benefit? Front. Educ. 7:875511. doi: 10.3389/feduc.2022.875511

[ref39] Stone S. A. (1984). Children's interpretations of wordless picture books (Ph.D. Dissertation.). Arkansas: University of Arkansas.

[ref40] Styles M. Arizpe E. (2001). A gorilla with 'grandpa's eyes': how children interpret visual texts—a case study of Anthony browne's zoo. Child Lit. Educ. 32, 261–281. doi: 10.1023/A:1012760422501

[ref41] Su M. (2021). A field study on Children’s book Reading reaction. Beijing, China: China Social Sciences Press.

[ref42] Sucena A. Silva A. F. Marques C. (2023). Reading skills promotion: results on the impact of a preschool intervention. Front. Educ. 7:1076630. doi: 10.3389/feduc.2022.1076630

[ref43] Terras M. M. Thompson L. C. Minnis H. J. D. (2009). Dyslexia and psycho-social functioning: an exploratory study of the role of self-esteem and understanding. Dyslexia 15, 304–327. doi: 10.1002/dys.386, PMID: 19384920

[ref45] Vezzani A. (2019). Conversation and learning in early childhood education: what works best for children’s cognitive development and how to improve pupil engagement? Eur. Early Child. Educ. Res. J. 27, 534–550. doi: 10.1080/1350293x.2019.1634240

[ref46] Wolfgang I. (1991). The act of Reading: A theory of aesthetic response. Beijing, China: China Social Sciences Press.

[ref47] Xie Q. L. (2007). Big class children's interpretation of picture books (Ph.D. Dissertation.). Zhengzhou: He'nan University

[ref48] Zhang X. (1993). Reader response theory and its enlightenment on Children’s literature education. Dongshi Chin J. 6, 268–307.

[ref49] Zheng H. (2011). Research on moral judgment among children aged 5–6 years old in picture book reading Master's Dissertation). Nanjing: Nanjing Normal University.

[ref50] Zhu Z. Q. (2008). Introduction to Children’s literature. Beijing: Higher Education Press.

